# Pancreatic Ductal Adenocarcinoma: Current and Emerging Therapeutic Uses of Focused Ultrasound

**DOI:** 10.3390/cancers14112577

**Published:** 2022-05-24

**Authors:** Maxime Lafond, Thomas Lambin, Robert Andrew Drainville, Aurélien Dupré, Mathieu Pioche, David Melodelima, Cyril Lafon

**Affiliations:** 1 LabTAU, The Institut National de la Santé et de la Recherche Médicale (INSERM), Centre Léon Bérard, Université Lyon 1, University Lyon, 69003 Lyon, France; andrew.drainville@inserm.fr (R.A.D.); aurelien.dupre@lyon.unicancer.fr (A.D.); david.melodelima@inserm.fr (D.M.); cyril.lafon@inserm.fr (C.L.); 2 Endoscopy Division, Édouard Herriot Hospital, 69003 Lyon, France; thomaslambin@hotmail.fr (T.L.); mathieu.pioche@chu-lyon.fr (M.P.)

**Keywords:** PDAC, focused ultrasound, HIFU, cavitation, drug delivery, sonodynamic therapy, immunotherapy

## Abstract

**Simple Summary:**

Pancreatic ductal adenocarcinoma (PDAC) is an increasingly prevalent form of cancer with a low patient survival rate following diagnosis. Focused Ultrasound is an emerging modality that provides exciting opportunities in treating PDAC. This review provides an overview of the clinical application and scientific research of therapeutic focused ultrasound for the treatment of PDAC for use by clinicians and scientific researchers. In addition to providing a description of various physical mechanism underlying therapeutic applications, the current benefits, challenges, and possible future avenues for the application and development of focused ultrasound in the treatment of PDAC are summarized.

**Abstract:**

Pancreatic ductal adenocarcinoma (PDAC) diagnosis accompanies a somber prognosis for the patient, with dismal survival odds: 5% at 5 years. Despite extensive research, PDAC is expected to become the second leading cause of mortality by cancer by 2030. Ultrasound (US) has been used successfully in treating other types of cancer and evidence is flourishing that it could benefit PDAC patients. High-intensity focused US (HIFU) is currently used for pain management in palliative care. In addition, clinical work is being performed to use US to downstage borderline resectable tumors and increase the proportion of patients eligible for surgical ablation. Focused US (FUS) can also induce mechanical effects, which may elicit an anti-tumor response through disruption of the stroma and can be used for targeted drug delivery. More recently, sonodynamic therapy (akin to photodynamic therapy) and immunomodulation have brought new perspectives in treating PDAC. The aim of this review is to summarize the current state of those techniques and share our opinion on their future and challenges.

## 1. Introduction

Pancreatic ductal adenocarcinoma (PDAC) is expected to become the second leading cause of mortality by cancer by 2030 [1]. Despite intensive research in the field of therapeutics, the 5-year overall survival remains around 5%, with only 20% of patients eligible for surgery at the time of diagnosis. Focused ultrasound (FUS) is a non-invasive therapeutic technique that uses the focalization of ultrasound waves to induce thermal or mechanical effect at the focal point. It should be noted that FUS usually refers to the broad range of exposure schemes, which includes the particular subset of high-intensity focused ultrasound (HIFU) specifically aimed at producing thermal effects by depositing energy at the target. To date, HIFU has primarily been used in gynecology and urology to manage uterine fibroma and prostate cancer, respectively, but clinical applications in PDAC are being studied extensively [2,3,4,5,6]; while initial studies have demonstrated efficacy in pain management, an increasing amount of data also suggest an anti-tumoral effect. Due to its effect on the tumoral microenvironment, FUS has been studied in combination with other therapies, such as chemotherapy or immunotherapy. HIFU is also being evaluated to facilitate local drug delivery. In this review, we will describe the different clinical applications of HIFU in PDAC management, how HIFU can impact the tumoral microenvironment, and the perspective of a combined approach to FUS to increase the efficacy of current anti-cancer drugs. After brief generalities on pancreatic cancer and the basic principles behind the therapeutic effects of focused ultrasound, the various uses of FUS in PDAC treatment will be covered, from the most advanced (current clinical use) to the most ongoing exploratory research.

## 2. Pancreatic Cancer Generalities

Pancreatic adenocarcinoma is a very lymphophyllic cancer that quickly becomes unresectable. Regardless of treatment, the overall 5-year survival rate for this disease is less than 5% and has shown only minimal improvement over the last few decades [7,8,9]. Most patients are treated with palliative intent due to either metastatic or locally advanced disease. When surgical resection is feasible, the 5-year survival rate is approximately 20%. However, surgery is possible in only 15–20% of patients [10,11]. Considering the high rate of unresectable tumors and the poor results of surgery alone in patients with pancreatic carcinoma, many treatment efforts incorporating chemotherapy, radiotherapy, or both have been made to improve the 5-year survival of these patients. As most patients (80–90%) are diagnosed with advanced (30–40%) or metastatic (50–60%) pancreatic carcinoma, the development of improved systemic treatment options has been a top priority over the last two decades. Patients with no metastatic disease but an unresectable tumor are defined as having locally advanced pancreatic adenocarcinoma (LAPA). This group represents 30–40% of patients. Treatment remains highly controversial, as it confers an average overall survival of only 9 to 14 months regardless of the treatment strategy. In this subgroup of patients, chemotherapy remains the standard of care. The combination of radiation therapy and chemotherapy is not recommended, as it has not demonstrated any survival advantages [12,13]. Unfortunately, most of these patients have a very limited chance of undergoing surgery even after chemotherapy [14]. In this context, pain management has an important place. A combination of endoscopic ultrasound-guided tumor ethanol ablation combined with celiac plexus neurolysis notably showed benefits in pain relief for a median duration of 10 weeks [15]. Given the high incidence of locally advanced pancreatic cancer and the low probability of downstaging with conventional treatment (chemotherapy and radiotherapy), there has been a growing interest in the use of new local ablative therapies, such as radiofrequency ablation (RFA) and irreversible electroporation (IRE), for multimodal treatment of the disease. These ablative techniques are applied to ultimately induce irreversible cellular damage to the tumor, leading to cell death via either apoptosis or coagulative necrosis [10,16,17]. Pancreatic neuroendocrine tumors (PNETs) represent a small fraction of all pancreatic tumors, with a better prognosis than PDAC (80% 5-year survival rate). The clinical data regarding the use of HIFU to treat PNETs are sparse and mostly consist of case studies [18,19]; while we acknowledge the work being performed on the use of HIFU in treating PNETs, the present review is limited to PDAC, which includes most of the clinical and preclinical studies.

## 3. Generalities on Focused Ultrasound

Focused ultrasound can induce bioeffects within deep tissue in a minimally or noninvasive fashion. The specific mechanisms underlying focused ultrasound therapy are usually divided into thermal and mechanical effects. Thermal effects are produced by the large amplitude, high duty cycle HIFU regime, and occur when the local tissue temperature rises above levels at which thermal necrosis occurs (>56 ${}^{{}^{\circ}}$C). The basic mechanism of tissue damage in HIFU thermal therapy is coagulative thermal necrosis [20]. As tissue temperatures rise above 43 ${}^{{}^{\circ}}$C, heating effects begin to produce protein denaturation and tissue damage. The rate at which thermal tissue damage accumulates can be predicted according to the Arrhenius equation, which exhibits a linear dependence on exposure time and an exponential dependence on tissue temperature [21,22]. Thermal dose for HIFU thermal therapy is typically quantified using equivalent time at 43 ${}^{{}^{\circ}}$C ($t_{43}$), which is given by [23]:

(1) $$t_{43}=\int_{0}^{t}R^{T(\tau )-43}d\tau ,$$

where *R* is 0.5 for temperatures above 43 ${}^{{}^{\circ}}$C and 0.25 below 43 ${}^{{}^{\circ}}$C, and *T(τ)* is the temperature as a function of treatment time, τ; while there is some variation depending on tissue type, thermal doses of 120–240 min at 43 ${}^{{}^{\circ}}$C typically lead to coagulation of critical cellular proteins and irreversible damage to tissue structural components and vasculature [21]. Above approximately 43.5 ${}^{{}^{\circ}}$C, the time required to produce a given effect is halved for each degree increase in temperature, with temperatures at or above 60 ${}^{{}^{\circ}}$C for 1 s leading to irreversible cell death in most tissues.

**Figure 1:**
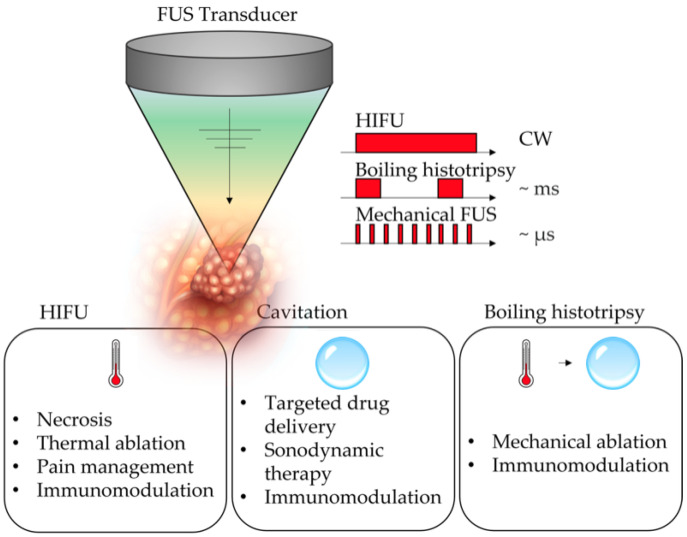
Schematic of the main ultrasound pulsing schemes used in FUS for PDAC treatment and their elicited bioeffects. CW indicates continuous wave.

Absorption of acoustic energy in fluids occurs due to frictional force, which acts to oppose the periodic vibration of molecules within the medium, leading to the production and accumulation of heat [24]. The ability to focus acoustic energy into a small volume allows for rapid and spatially confined heat generation at the target while minimizing effects in surrounding tissues. For short exposure times, the increase in temperature is largely confined to the focal region, while for longer exposures, thermal conduction and blood circulation diffuse heat into the surrounding tissues, resulting in a more diffuse temperature distribution [25]. At high acoustic intensities, nonlinear wave propagation causes high-pressure portions of the wave to travel faster than lower-pressure portions, leading to a distortion of the temporal waveform and production of higher harmonics of the fundamental acoustic frequency. Sufficient nonlinear propagation may give rise to shock formation within tissues, which are capable of heating tissue much more rapidly than would be expected from frictional absorption alone. This rapid increase in tissue heating can lead to boiling and production of bubbles within a few milliseconds [26,27]. The strong acoustic contrast between the gas bubbles and surrounding tissue causes strong reflection and scattering of acoustic waves, with backscattered waves that may interact with incident acoustic waves and stimulate further bubble growth, causing a rapid change in the acoustic dynamics and modification of the ultrasound field [28]. The rapid explosion of small boiling bubbles and their cascading interactions with shocks can cause mechanical fractionation and emulsification of tissues without inducing thermal damages in surrounding tissues [27,29,30]. Mechanical effects encompass a variety of phenomena, including cavitation (stable and inertial), radiation force, and acoustic streaming. These effects are more often induced using high-pressure, short-duration ultrasound pulses, which allow for high instantaneous intensity without the accumulation of thermal energy and production of thermal effects. Numerous methods rely on mechanical effects, including ultrasound microbubble targeted destruction (UMTD), histotripsy, lithotripsy, sonodynamic therapy, sonothrombolysis, and blood–brain barrier opening [20,29,31,32,33,34,35,36,37,38]. Acoustic cavitation is a nonlinear mechanical phenomenon that arises from the interaction of a gas bubble with the oscillating pressure field [39], and therefore, requires the presence of a nucleating bubble site within the tissue, which may include sub-micrometer gas bodies that exist naturally in vivo [40,41,42]. Acoustic cavitation is usually classified as either stable or inertial, even though any cavitation cluster will present a mix of those. At relatively low intensities, the bubble will undergo stable (or non-inertial) cavitation, where the bubble oscillates around its equilibrium radius, with displacement of the surrounding medium, and growing with rectified diffusion [43,44]. Inertial cavitation occurs when acoustic wave amplitudes are high enough that the medium displaced by the bubble expansion has enough momentum in the compression phase to drive the bubble to a violent collapse, which can lead to the generation of extremely high temperatures and pressures, as well as shockwave generation [45,46] and mechanical damage to tissues, as is observed in histotripsy [38,47,48]. The cavitation threshold is mainly driven by the frequency, the peak negative pressure, and the physical parameters of the medium [40,41,49,50]. In the case of ultrasound contrast agents, their rheological properties also have an impact on the cavitation activity [51]. Figure 1 summarizes the US regimen, as well as the bioeffects typically elicited in clinical and preclinical studies on FUS and HIFU in PDAC treatment.

## 4. Current Clinical Applications of HIFU in the Pancreas

**Figure 2:**
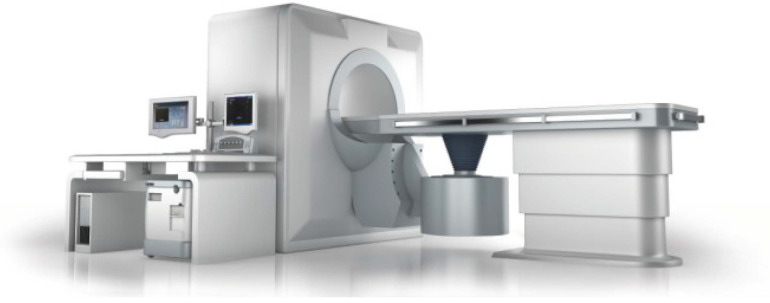
Haifu clinical HIFU system.

HIFU has been demonstrated to reduce pain associated with pancreatic cancer since the early 2000s, which is currently the main clinical application of FUS that is part of PDAC management. Typical cases of HIFU treatments include target visualization, tissue change monitoring, and treatment outcome assessment, all of which can currently be achieved with either using MRI or diagnostic US on commercially available systems, such as the widely used Haifu system (Figure 2). A prospective study by Wang et al. [52] evaluated the ability of HIFU to reduce pain in 40 patients with unresectable PDAC. Among them, 87.5% experienced partial or complete pain relief after HIFU treatment, with a median pain relief time of 10 weeks. Similarly, in a prospective European study, among 50 patients with late stage pancreatic cancer, 84% of the patients had a reduction of pain within the first week following the procedure, which persisted over time, and for 50% of patients, analgesic medication was no longer necessary at 6 week [53]. The mechanism of pain relief may be due to a reversible block of nerve activity [54]. A multicenter study among two European centers evaluated the impact of HIFU on quality of life in 80 patients with an inoperable pancreatic cancer. Nearly half of them had a metastatic disease and nearly all of them had an ECOG (Eastern Cooperative Oncology Group) functional status of 0 or 1. A majority of patient were treated by one session of HIFU, with 10% of the patients having two sessions. Quality of life was evaluated by a questionnaire (EORTC questionnaire). Global health status was significantly increased 3 and 6 months after HIFU treatment with improvement of physical, role, and emotional functioning at 6 months and of emotional and social functioning at 3 months. Effects were independent of tumor stage, metastatic status, or country [55]. HIFU has also been described to allow local tumoral control. A study by Marinova et al. [3] evaluated the effect of HIFU treatment on 13 patients with PDAC (5 stage III, 8 stage IV UICC disease), 10 of whom received chemotherapy. Following HIFU, contrast-enhanced ultrasound (CEUS) revealed devascularized tissue (no contrast enhancement). Tumor volume did not change significantly within the first week following the procedure—however, tumor volume at 3 months regressed considerably (63% reduction). Similar results were obtained by Strunck et al. [5] in 15 patients with locally advanced inoperable pancreatic cancer. A study by Wang et al. [56] on 30 patients evaluated the impact of HIFU treatment as a preoperative adjuvant therapy for borderline resectable pancreatic cancer. The mean tumor ablation rate was 61% in the 30 patients, with 28 patients undergoing surgical resection of cancer around 7 weeks after HIFU treatment. The R0 resection rate was 92.7% with a 1-year survival rate of 96.7%. A systematic review evaluated the efficacy of HIFU combined with other treatment modalities, in which 23 studies were included. The survival rate at 6 months and 12 months, overall efficacy, and clinical benefit for patients undergoing HIFU combined with radiation and chemotherapy was significantly higher than radiation therapy or chemotherapy (gemcitabine, gemcitabine + cisplatin, gemcitabine 5-fluorouracil). However, the quality of these studies was relatively low as per the Oxford Centre for Evidence-Based Medicine criteria [57]. HIFU was also used as a complementary treatment of distal biliary obstruction secondary to pancreatic cancer with self-expended metallic stent (SEMS). In a retrospective study, the clinical efficacy and long-term outcomes of SEMS combined or not with HIFU ablation were evaluated in 75 patients with distal biliary obstruction secondary to PDAC [58]. A total of 34 patients were treated by a SEMS alone and 41 by SMES and HIFU ablation. Median stent patency was significantly longer in the SEMS with HIFU group, with 175 days versus 118 in the SEMS-only group. The median survival time was significantly longer in the stent + HIFU group with 211 days versus 136 days in the stent only group. Predictors of prolonging survival were ECOG performance status of 3 and HIFU ablation treatment [58].

## 5. Thermal Effects of HIFU in PDAC

Thermal ablation has been studied vastly in pancreatic cancer [59]. Structural and functional changes to tumoral blood vessels within pancreatic tissues results in irreversible decrease in tumor blood flow, reducing the local cooling effects of blood perfusion and leading to heat trapping and progressive tissue damage [60]. A reduction of the blood supply associated with thermal therapy may lead to oxygen and nutrient deprivation, enhancing overall tissue destruction. The boundaries of thermal lesions are typically defined by the 50–54 ${}^{{}^{\circ}}$C contour and a $t_{43}$ dose greater than 240–540 min. At lesion borders or regions where immediate cell death is not induced, the phenomenon of thermal fixation may occur, where lower but still lethal thermal exposure is present, leaving tissue architecture intact and leading to cell death typically within 2–3 days [22,61]; while lysis of pancreatic cells has potential to release autodigestive enzymes, leading to pancreatitis, pancreatic cells that experience thermal fixation will not undergo lysis until the intracellular enzymes have been completely denatured and inactivated, which may reduce the risk of pancreatitis with HIFU therapy [61,62]. The feasibility of intraoperative HIFU ablation of pancreatic parenchyma has been demonstrated in porcine models without severe acute pancreatitis or serious intra-abdominal complications [63], including the use of ultrasound imaging for treatment guidance and evaluation of treated regions [64].

The potential for HIFU treatment for the treatment of malignant pancreatic tumors has been demonstrated in clinical studies by Vidal-Jove et al. [65], where a significant and clinically meaningful survival advantage was observed in 43 patients with unresectable pancreatic tumors treated with ultrasound-guided HIFU ablation in combination with adjuvant chemotherapy. This study also demonstrated that for patients with stage III tumors and minimal vascular invasion who are not candidates for surgical resection, HIFU may provide a curative treatment to achieve long-term disease-free survival.

The increased sensitivity of cancer cells to thermal stress—attributable to higher metabolic stress, lower thermal conductance, and a lower cancer microenvironment pH—has made thermal ablation methods attractive for treatment of solid parenchymal tumors. Locoregional thermo-ablative techniques present lower rates of morbidity, better preservation of surrounding tissue, shorter hospital stays, and overall lower cost compared to surgical intervention; while the application of thermal ablation techniques for pancreatic tumors has previously been limited due to the risk of severe complications caused by injury to the pancreatic parenchyma and surrounding structures, interest has been growing in the applications of these techniques for the treatment of PDAC [66].

Numerous studies have shown HIFU to be a safe and effective means of pain relief in patients with pancreatic cancer. A meta-analysis of the use of HIFU for the palliative treatment of pancreatic cancer estimated that 80% of patients experienced partial or total pain relief following HIFU treatment, while 74% of patients had a positive tumor response [2,67]. Tumor size reduction does not appear to be a sensitive metric to evaluate HIFU efficacy for pain reduction or overall ablation success, as reduction in pain may occur without a decrease in volume, or even with an increase in volume due to local edema. The mechanisms by which HIFU may produce pain relief are not currently well understood and may be attributable to damage to the nerve fibers innervating the tumor or the abscopal effect due to immunomodulation [61,62]. These findings suggest that HIFU is an effective means of relieving pain in patients with pancreatic cancer, with low risks of adverse events [2]. The most common side-effects or complications are skin burns at the application site (10% of the patients) and osteonecrosis along the ultrasound beam path (7%). HIFU reportedly induced mild and transient pain after exposure in 15% of the patients. More rare complications include pancreatitis (6%) and pleural effusion (2.5%). Cholecystitis, biliary tract obstructions, renal impairment and hematuria, supraventricular tachycardia, hypertension, and liver abscesses were also reported (<2%) [54].

Several studies suggest that HIFU thermal ablation may elicit a systemic antitumor immune response, though possible mechanisms are still unclear. Hypothesized methods based on previous results include reduced host immune suppression, modification of antitumor antigenicity and upregulation of HSP proteins, cytokine secretion at inflammatory margin of ablation treated regions stimulating the development of mature cytotoxic T-cells, and large amounts of cellular debris that are phagocytized by macrophages and other cells that can function as antigen-presenting cells [60].

Recent studies have demonstrated that HIFU thermal therapy may have a synergistic effect when used in combination with chemotherapy, with an increase in tumoral drug concentration and reduced systemic toxicity. These effects are believed to be due to increased endothelium permeability and enhanced diffusion of the chemotherapeutic agent caused by radiation force of the acoustic field [2,61]. Studies have notably highlighted a positive impact of HIFU ablation on survival rates [57,68,69,70]. Although these are encouraging studies, the low methodology level, as per the Oxford Centre for Evidence-Based Medicine criteria [71], demands further investigations to assess the potential impact on patient survival.

## 6. Mechanical Effects of FUS in PDAC

In 2015, Li et al. [57] showed, in KPC mice, that cavitation with pulsed FUS enhanced the intratumoral concentration of doxorubicin 4.5-fold compared to controls, with an increase of doxorubicin concentration when cavitation was high and sustained. There was no difference when pulsed HIFU was delivered during or before doxorubicin administration. For the pulsed HIFU-treated tumors, macroscopic evaluation revealed hemorrhagic areas, and microscopic evaluation showed disorientation and separation of the collagen matrix with fraying of collagen fibrils [57]. A study from our group evaluated the impact of various inertial cavitation regimen combined with gemcitabine on the viability of PDAC spheroids composed of both KPC pancreatic cancer cells and activated fibroblasts designed to mimic the tumor stroma [72]. This model possessed some of the essential features of PDAC, including the presence of activated fibroblasts, production of extracellular matrix, and a dense intracellular arrangement. Inertial cavitation was shown to decrease the viability of spheroids when exposed to cavitation and gemcitabine, compared to cavitation or gemcitabine alone. Moreover, gemcitabine had no impact on fibroblast viability, whereas the effect of chemotherapy on PDAC cell viability was enhanced when combined with cavitation. Importantly, the toxicity of gemcitabine was less important on spheroids composed of both KPC cells and fibroblasts compared to those composed of KPC cells only, which is consistent with the protective effect of TME and supports the benefit of the combination. However, studies show that fibroblasts also act to limit tumor growth by restraining angiogenesis, and that fibroblast depletion could accelerate tumor progression [73,74]. Finding balance between increased drug penetration and intact angiogenesis restriction might become a challenge in the short-term future of this approach.

Huang et al. [75] evaluated the impact of cavitation induced with an ultrasound contrast agent (microbubbles) in a mouse model of pancreatic cancer. Blood perfusion evaluated by contrast-enhanced ultrasound imaging revealed a decrease of blood flow within the tumor after cavitation treatment compared to pre-treatment measurement, whereas blood perfusion of non-tumoral tissue was not impacted. Immunostaining of blood vessels also showed a decreased expression of CD31 in the cavitation group, with a reduced microvascular density.

The clinical data on the combination of FUS and chemotherapy for treating PDAC are scarce. In a phase I clinical trial, 10 patients were enrolled to receive gemcitabine combined with low-intensity ultrasound using microbubbles as an ultrasound contrast agent programmed to favor sonoporation, with encouraging results in terms of the number of chemotherapy cycles tolerated and experienced median overall survival when compared to 63 historical controls receiving only chemotherapy [76]. An upcoming phase II randomized clinical trial (NCT04146441) of FUS combined with chemotherapy (FOLFIRINOX) and microbubbles (SonoVue) will aim to determine whether FUS can increase drug uptake and overcome chemoresistance in a study with 30 patients. HIFU is a very attractive way to increase intratumoral temperature and increase drug delivery. In a monocentric retrospective study among 523 patients, a combination of HIFU with gemcitabine appeared to achieve a better overall survival than standard chemotherapy in unresectable PDAC [77].

## 7. FUS-Mediated Targeted Delivery

Focused ultrasound can be used to selectively deliver chemotherapy within the pancreatic tumor using micron-sized loaded carriers. These carriers are bio-compatible and are designed to encapsulate chemotherapy to protect healthy tissue from off-target effects. The most-used particles are liposomes and micelles. Once the particle has reached the tumor, it is triggered to release its contents. This controlled release can be produced through several mechanisms, including FUS [78]. Hyperthermia from HIFU can cause the temperature to reach a level at which pores appear within the bilayer of the liposome, allowing the release of chemotherapy. Alternatively, the shock waves produced by cavitation close to the bilayer membrane can cause it to open. Moreover, cavitation can induce formation of microjets that can puncture tumoral cells if the jet is directed towards them, thereby enhancing the effect of FUS targeted therapy with liposome [78].

A study by our group evaluated the efficacy of a combined treatment with inertial cavitation and liposomal DOX (L-DOX) on an orthotopic model of pancreatic cancer in mice and rats [79]. After a follow-up at 9 weeks, the group treated with combined US and L-DOX exhibited significantly lower tumor volumes than the sham group, the US group, and the L-DOX group, with a trend towards lower tumor volume compared to the group treated with gemcitabine, which was not statistically significant. No difference was observed in rats, most likely due to tumor implantation issues, according to the authors. A similar encapsulation strategy using gemcitabine instead of DOX may be more efficient.

Another study by Farr et al. [80] evaluated the enhancement of drug delivery in KPC mice (genetically engineered mouse model of PDAC) with targeted mild hyperthermia generated by magnetic resonance guided HIFU (MRgHIFU) treatment in combination of a low-temperature sensitive liposomal formulation of DOX (LTSL-DOX), which produced a 23-fold increase of the localized drug within the tumor tissue compared to LTSL-DOX. When HIFU was combined with a regular form of DOX, hyperthermia produced only a 2-fold increase in drug concentration compared to DOX alone [80].

The Pandox study is an ongoing clinical trial that aims to determine the intratumoral concentration of doxorubicin when delivered with ThermoDox (an encapsulated thermally sensitive liposomal form of DOX) combined with mild hyperthermia generated by focused ultrasound compared to the free drug alone among 18 patients with a non-resectable or metastatic PDAC (NCT04852367).

Targeted drug delivery can also be achieved by other means. In a pancreatic cancer xenograft mouse model, Kang et al. evaluated the effect of a DOX-loaded microparticle-microbubble complex (DMMC) combined with pulsed FUS. Compared to DMMC only, DOX only, DOX+US, and non-treated, the group treated with combined DMMC and FUS had the smallest tumor size at 4 weeks, the smallest growth rate at 4 weeks, and a greater intratumoral DOX released [81]. The mechanism of action could be the following: the DMMC arrives to the tumor site, and the DOX microparticle is dissociated from the microbubble with the action of FUS. In parallel, FUS allows the generation of inertial cavitation of the microbubbles, leading to a extravasation of the drug through the tumor vessels, which then enters tumors cells after an increase of cell membrane permeability generated by FUS [81,82,83]. Similarly, in rats bearing one orthotopic tumor at each flank, sonication of injected DOX-loaded phospholipid microbubbles (mechanical index of 1.6 at 1.3 MHz) on one side led to a 12-fold concentration of intratumoral DOX and reduced growth compared to the side not exposed to [84]. The results are summarized in Table 1.

## 8. Sonodynamic Therapy

Sonodynamic therapy (SDT) consists of the synergetic action between sound and a chemical agent, usually to trigger the release of reactive oxygen species (ROS) to induce cellular damage [85,86,87]. SDT partly relies on intratumoral oxygen availability, which is a specific challenge in the pancreas due to the intrinsic hypoxia in PDAC. Carbon-coated titanium dioxide TiO${}_{2}$/C nanocomposite addresses this challenge, with the ability to produce ROS in an oxygen-independent manner. This sonosensitizer demonstrated increased damage to tumor cell DNA and a reduction in subcutaneous Panc02 tumor growth in BALB/C mice [88]. Another approach is to deliver oxygen to the tumor vicinity to increase its partial pressure, either by intravenous injection of oxygen-loaded microbubbles (O${}_{2}$-MB) or using oral oxygen nanobubbles [89,90]. In combination with Rose Bengal, a common sonosensitizer, O${}_{2}$-MB reduced the growth of BxPc-3 ectopic tumors in mice compared to SF6-MB [91]. Such treatment was further improved by the addition of antimetabolites such as gemcitabine [92] or 5-fluorouracil [93]. This last study interestingly showed evidence of immunomodulation following SDT through a decreased expression of Bc13. The same group further refined their platform agent by adding a magnetic feature to it, which helped to retain the MBs in the tumor vicinity and increase apoptosis [94]. Additionally, they used a chemo-sonodynamic complex comprising gemcitabine and Rose Bengal, linked with biotin, and demonstrated the superiority of this technique over the use of separate compounds in murine BxPC-3 xenografts [89]. The potential of antimetabolites was confirmed by Browning et al. in a study showing the growth reduction in PSN-1 and BxPC-3 pancreatic tumor models [95]. However, improved survival was only achieved in the PSN-1 model. The authors hypothesized that SDT achieves better results in poorly vascularized tumors. NC-6300 is a compound with a pH-dependent release of epirubicin. NC-6300 naturally accumulates in the tumor due to the enhanced permeability and retention (EPR) effect, and the acidic condition of the tumoral microenvironment induce the release of epirubicin. A sonodynamic effect was demonstrated in subcutaneous MIA PaCa-2 tumors in mice [96]. However, due to its limited range of action within the tumor, SDT should not be pursued as a standalone therapy, but in combination with other conventional treatment strategies [97], such as photodynamic therapy, which demonstrated efficacy in combination with radiotherapy in clinical settings [98,99]. It should be highlighted that SDT recently showed evidence for influencing the immune response in pancreatic cancer models, which provides exciting perspectives for the technique [100,101]. The results are summarized in Table 2.

## 9. Immunotherapy

FUS has been shown to improve the immune response against tumors since the 2000s [102,103,104]. The mechanisms for the enhancement of the immune response are numerous and beyond the scope of this paper. It is accepted that subcellular fragmentation produced in situ by US are subsequently presented to dendritic cells [105] and trigger cytotoxic T cell activation [106]. Note that T-cell activity in PDAC is greatly reduced by the hypovascularized, acidic, and hypoxic microenvironment. As tumor hypoxia promotes immunosuppressive activity [107,108], relieving the hypoxic stress could enhance the endogenous immune response in addition to improved response to radio- and chemotherapy. Pulsed FUS or low-intensity FUS have been shown to drive Th1 inflammation, to stimulate localized cell recruitment factors and tumor cell surface immunogenic proteins, and also to increase CD8+/T regulatory cell ratio [109]. However, these data come from non-PDAC tumor types. The number of studies investigating ultrasound on the immune response has expanded wildly in the last few years, even though most involve non-pancreatic tumors. In the pancreas, an early study using thermal ablation reported modulation of the immune response [103]. More recently, a retrospective study also reported immunomodulation and reduced tumor volume after a one-year follow-up subsequently to HIFU thermal ablation [110]. More anecdotally, two case studies reported abscopal effects following thermal ablation [111,112]. Preclinical data regarding immune effects in animal models of pancreatic cancer have recently been published [100,101,113,114]. The interested reader can refer to the detailed comprehensive review of those studies recently provided by Mouratidis and ter Haar [115]. Currently, there is a good indication that mechanical and thermal effects of ultrasound can modulate the immune response, and that this area of research should be pursued with the goal of creating strong and durable immune response to pancreatic cancer.

## 10. Conclusions and Future Directions

The reduction in tumor volume, retraction of tumor from involved vessels, and downstaging show that HIFU potentially will have comparable results to standard neoadjuvant treatment options. Future areas of research should be aimed at investigating the effects of neoadjuvant treatment with chemotherapy in association with HIFU, comparison of median survival rates of patients, rates of achieved R0 resections, and differences in complication rates after surgery by randomized trials and prospective control-cohort studies. Multidisciplinary team decisions between surgeons, oncologists, HIFU specialists, and radiologists could prove useful in the decision making for optimal neoadjuvant treatment. HIFU is found to be safe and feasible in locally advanced and metastatic pancreatic cancer with proven downstaging and downsizing effects. Further research on role of HIFU ablation as a neoadjuvant treatment for borderline resectable pancreatic cancer is needed. The mechanical effects of FUS, and mostly cavitation, have been widely studied in an effort to enhance the penetration of chemotherapy. It seems that the key point is how the drug crosses the dense tumor stroma. This hypothesis meets a serious obstacle as studies have reported that this stroma also acts to hinder tumor progression, so its disruption may not necessarily be beneficial in all cases. Better characterization of the tumor mechanical properties (e.g., using elastography) might give insight on the expected tumor response to the mechanical effects of FUS. Moreover, recent breakthroughs in localizing [85,116], imaging [117,118,119,120,121,122], controlling [123,124], and quantifying [125,126,127] cavitation activity will most certainly improve the level of control of those mechanical effects, increase reliability, and allow fine tuning of the US parameters; while a limited amount of clinical data are currently available regarding drug delivery to pancreatic cancer, the increasing body of preclinical data and the use of ultrasound in drug delivery and other therapeutic applications suggest an increase in clinical studies being conducted in the near future.

Sonodynamic therapy and immunotherapy are currently more exotic application of US. Although those techniques present tremendous opportunities, they should be considered cautiously in light of the current lack of clinical data. Sonodynamic relies on sonosensitive agents, most of which happen to be photosensitive (the role of sonoluminescence is frequently discussed in SDT literature) and approved for photodynamic therapy, which gives SDT a head start toward clinical testing. On the other hand, functionalized platforms that include multiple agents (SDT agent, magnetic particle, molecular targeting, bioactive gases such as oxygen, etc.) are promising to attack the tumor from multiple angles, but their clinical approval might be difficult to obtain in the short term. Immunotherapy is arguably the fastest growing application of therapeutic ultrasound. Initial clinical and preclinical data are astounding and the possibility of abscopal effects covers for FUS’ blind-spot: treatment of metastases. More studies are required to produce a robust and sustained immune response. Overall, those techniques and use of FUS are not competing, as there is no contraindication in combining multiple modalities, notably with mechanical effects or SDT and immunotherapy.

## Figures and Tables

**Table 1:** Studies on FUS-mediated targeted drug delivery in pancreatic tumor models.

Year	Tumor Model	Targeting Agent	Focused Ultrasound Parameters	Results	Ref
2010	Orthotopic pancreatic cancer in Lewis rats	DOX-loaded phospholipid microbubbles	1.3 MHz, MI 1.6	12-fold increase in intratumoral DOX	[84]
2018	KPC (mouse)	Low-temperature sensitive liposomes loaded with doxorubicin (LTSL-DOX)	Clinical MR-HIFU system (Sonalleve V1, Philips, Vantaa, Finland). 1.2 MHz, 10 acoustic power, 20 s duration, continuous wave.	Induced mild hyperthermia. HPLC and fluorescence microscopy demonstrated a 23-fold increase in intratumoral DOX compared to LTSL-DOX alone	[80]
2019	orthotopic DSL6A/C1 pancreatic cancer in 5-week-old male Lewis LEW/CrlBR rats, and MIA PaCa2 orthotopic cancer in 4-week-old nude mice (NMRI-Foxn1nu/nu)	Sonosensitive liposomal DOX (L-DOX)	In the rats: 1.1 MHz, 6/5.85 W average electrical power, 200/250 Hz PRF, 0.77/1.00% duty cycle. (rats/mice)	Reduced tumor growth in US+L-DOX group compared to L-DOX in mice only	[79]
2020	Immunodeficient mice inoculated with CFPAC-1 cells	DOX-loaded microparticle-microbubble complexes (DMMC)	Preclinical FUS system (VIFU2000, Alpinion, Seoul, Korea), 1.1 MHz, 14.8 MPa PPP, 9.2 MPa PNP, 40 Hz PRF, 5% duty cycle, 800 pulses (20 s total duration)	Reduced tumor growth in US+L-DOX group	[81]
Ongoing	18 patients with PDAC enrolled	Heat-sensitive chemotherapy drug (ThermoDox, Celsion Corp.)	Subablative levels	NA	Unpublished (PanDox clinical trial ongoing: NCT04852367)

**Table 2:** Studies on sonodynamic therapy in pancreatic tumor models.

Year	Tumor Model	Sonosensitive Agent	Focused Ultrasound Parameters	Results	Ref
2021	BxPC-3 xenografts in mice	Oral oxygen nanobubbles, RB intratumoral injection	1 MHz, 0.1 kHz PRF, 30% duty cycle, 3.5 W/cm², 3.5 min	Reduced tumor growth in groups receiving oxygen bubbles 5 or 20 min before SDT. Changes in tumor ocygen levels confirmed following tumor excision	[90]
2015	BxPC-3 xenografts tumors in mice	O${}_{2}$MB-RB conjugates	Sonidel SP100, 1 MHz, 3.0 W/cm² ISATP, 30% duty cycle, 100 Hz PRF, 3.5 min	Reduced tumor growth with O${}_{2}$MB-RB compared to SF6MB-RB	[91]
2016	BxPC-3 xenografts tumors in mice	O${}_{2}$MB-5FU or O${}_{2}$MB-RB	1 MHz, 3.5 W/cm² ISATP, 30% duty cycle, 100 Hz PRF, 3.5 min	Reduced tumor growth with O${}_{2}$MB-RB/O${}_{2}$MB-5FU mix + US compared to O${}_{2}$MB-RB + US and controls. Plausible immunomodulation through Bcl3 downregulation.	[93]
2017	BxPC-3 xenografts tumors in MF1 mice	MagO${}_{2}$MB-RB and MagO${}_{2}$MB-5FU	1 MHz, 3.5 W/cm² ISATP, 0.85 MPa peak-peak, 30% duty cycle, 100 Hz PRF, 3.5 min	Tumor growth reduced significantly when the magnetic field was turned on, and not significantly when it was turned off	[94]
2018	MIA PaCa-2 xenografts in SCID mice	O${}_{2}$MB-Gem, O${}_{2}$MB-RB	Sonidel SP100, 1 MHz, 3.5 W/cm², 0.48 Mpa PNP, 30% duty cycle, 100 Hz PRF, 3.5 min	Tumor growth delay using the O${}_{2}$MB-Gem/O${}_{2}$MB-RB conjugates	[92]
2020	BxPC-3 xenografts in mice	Oxygen-loaded magnetic microbubbles (MagO${}_{2}$MBs) and Rose Bengal-gemcitabine chemo-sonodynamic complex	1.17 MHz, 100 Hz PRF, 30% DC, 0.7 MPa PNP, 3.5 min	Decreased tumor size in the folowing days. RB+Gem complex was more efficient than the separate compounds	[89]
2021	PSN-1 and BxPC-3 pancreatic tumors in female Crl:NU(NCr)-Foxn1nu mice	O${}_{2}$MB-RB + Chemo-radiotherapy (Gem + 4 Gy)	Sonidel SP100, 1 MHz, 3.5 W/cm², 0.88 Mpa PNP *, 30% duty cycle, 100 Hz PRF, 3.5 min	Improved survival in PSN-1 model only	[95]
2017	MIA PaCa-2 xenografts in male CAnN.Cg-Foxn1 nu/CrlCrlj mice	NC-6300: releases epirubicin in the acidic tumoral microenvironment	1.09 MHz, Bimodal excitation: 8 kW/cm² 20-ms pulses at 100 Hz PRF intercalated with 360 or 270 W/cm² 9.98-ms pulses.	Tumor growth inhibition	[96]
2021	Bilateral T110299 xenografts in C57BL/6JOlaHsd mice	O${}_{2}$MB-RB + anti-PD-L1 checkpoint inhibitor	1 MHz, 3.5 W/cm², 0.48 Mpa PNP, 30% duty cycle, 100 Hz PRF, 3.5 min	Reduced tumor growth. Immunomodulation observed following SDT (abscopal effect)	[100]
2021	BxPC-3 xenografts tumors in SCID mice and bilateral T110299 xenografts in C57BL/6JOlaHsd mice	RB-loaded, pH-sensitive polymethacrylate-coated CaO${}_{2}$ nanoparticle	Sonidel SP100, 1 MHz, 3 W/cm${}^{2}$ ISATP, 30% duty cycle, 100 Hz PRF, 3.5 min	Reduced tumor growth. Immunomodulation (abscopal effect) observed following SDT in the C57BL/6JOlaHsd mice	[101]
2021	Subcutaneous Panc02 in female BALB/C mice	Carbon-coated tutanium dioxide nanocomposites (TiO${}_{2}$/C)	1 MHz, 0.5 W/cm², 50% duty cycle, 1 min duration, repeated 1, 2, or 3 times. Pulse duration was not indicated in the study	Increased damage to cell DNA, growth reduction. Efficacy was function of the number of US exposures	[88]

* indicates a parameter where reporting error is suspected based on similar studies from the same group with the same equipment.

## Abbreviations

The following abbreviations are used in this manuscript:

PDAC	Pancreatic ductal adenocarcinoma
US	Ultrasound
HIFU	High Intensity Focused Ultrasound
FUS	Focused ultrasound
LAPA	Locally advanced pancreatic adenocarcinoma
RFA	Radiofrequency ablation
IRE	Irreversible electroporation
UMTD	Ultrasound microbubble targeted destruction
ECOG	Eastern Cooperative Oncology Group
EORTC	European Organisation for Research and Treatment of Cancer
UICC	Union for International Cancer Control
CEUS	Contrast-enhanced ultrasound
SEMS	Self-expanding metal stents
HGU	Hepatic glucose uptake
HSP	Heat shock protein
CTL	Cytotoxic T lymphocyte
TME	Tumoral microenvironment
MRgHIFU	Magnetic resonance guided high intensity focused ultrasound
DMMC	DOX-loaded microparticle-microbubble complex
